# Supplementary material for: Electro-Optical Sampling of Single-Cycle THz Fields with Single-Photon Detectors
Source: Sensors (Basel). 2022 Dec 2;22(23):9432. doi: 10.3390/s22239432 (PMC9739430; doi:10.3390/s22239432)
Supplement: Supplementary file 1 [file sensors-22-09432-s001.zip › sensors-1984595-supplementary.pdf]

Supplementary Materials:

Electro-optical sampling of single-cycle THz fields with single-photon detectors

Taylor Shields[1,*], Adetunmise C. Dada[2], Lennart Hirsch[1], Seungjin Yoon[1], Jonathan M. R. Weaver[1], Daniele Faccio[2], Lucia Caspani[3], Marco Peccianti[4], Matteo Clerici[1]

[1] James Watt School of Engineering, University of Glasgow, Glasgow G12 8QQ, UK
[2] School of Physics and Astronomy, University of Glasgow, Glasgow G12 8QQ, UK
[3] Institute of Photonics, Department of Physics, University of Strathclyde, Glasgow G1 1RD, UK
[4] Emergent Photonics Research Centre, Department of Physics, Loughborough University, Loughborough LE11 3TU, UK

S.1. THz Knife-edge Measurement

The THz beam profile $G(x,y) = \exp\left(-x^2/\sigma_x{}^2 - y^2/\sigma_y{}^2\right)$ in the focus of the detection crystal was measured using a knife-edge measurement where a sharp edge is used to incrementally interrupt the THz beam until the voltage output across a pyroelectric detector reduces to zero. The typical curve obtained using a knife-edge method relies on the standard Gaussian error function (erf) well documented in the literature for this method [1]. The estimated Gaussian width of the THz spot size in the focus of the parabolic mirror in the x direction was $\sigma_x = 200 \pm 10$ µm at the $1/e^2$ condition. Identical results were obtained for the measurement along the y direction, i.e., $\sigma_x \simeq \sigma_y$.

S.2. Statistical properties of the squeezed vacuum

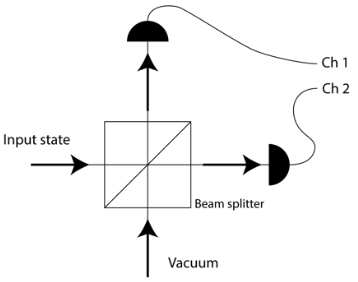

Figure S1: Beam splitter geometry.

In the quantum theory of light, a detector is assumed as the photon-number operator n̂, which resolves the photon number. However, most single photon counters are not photon number resolving (PNR) detectors but rather on/off detectors. As opposed to the PNR case, on/off sensors are only able to distinguish a zero photon case $|0\rangle$ from a multiphoton one $|n = 1, 2, 3, \dots\rangle$. Therefore, the statistical properties retrieved from on/off measurement require a suitable analysis.

When an on/off detector measures an incoming photon state, there are two events: on and off. The probability of an *off* result is $P(\text{off}) = P(0) = |\langle 0|\psi\rangle|^2$, where $|\psi\rangle$ is the quantum state measured [2]. The probability of the *on* case is obtained from the former considering: $P(\text{on}) = 1 - P(\text{off})$. Using this relation, the statistical properties of the states can be calculated as follows:

$$\bar{n}_{\text{on/off}} = 0 \times P(\text{off}) + 1 \times P(\text{on}) \tag{S1}$$

$$\sigma^2(n)_{\text{on/off}} = \left(0 - \bar{n}_{\text{on/off}}\right)^2 \times P(\text{off}) + \left(1 - \bar{n}_{\text{on/off}}\right)^2 \times P(\text{on}) \tag{S2}$$

Considering the input state impinging on a beam splitter as in Fig. S1, the variance of the differential measurement between Ch1 and Ch2, $\sigma^2(n^-)_{\text{D}}$ can be written as

$$\sigma^2(n^-)_{\text{D}} = \sigma^2(n_1)_{\text{D}} + \sigma^2(n_2)_{\text{D}} - 2\text{Cov}(n_1, n_2)_{\text{D}}\,, \tag{S3}$$

where D is the detection method (on/off or PNR), $\sigma^2(n_i)_{\text{D}}$ is the variance of the measurement on each channel i, and $\text{Cov}(n_1, n_2)_{\text{D}}$ is the covariance between the two channels. The covariance depends on the correlations of the input state. Considering a squeezed vacuum as input states, for on/off detectors, it holds

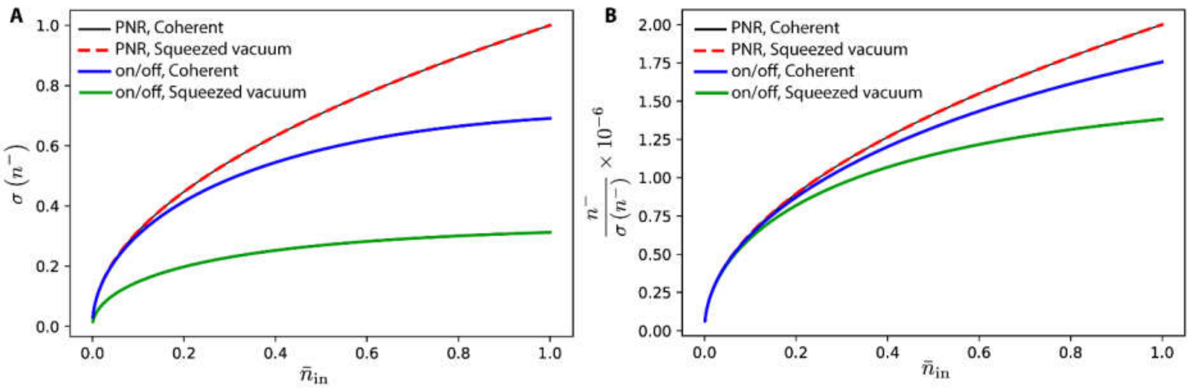

Figure S2: Theoretical analysis of the standard deviation and sensitivity. **A**: Theoretical standard deviation $\sigma(n^-)$ of differential measurements using on/off and photon number resolving detectors as a function of the average number of photons per pulse in the input state, $\bar{n}_{\text{in}}$, for a 50:50 beam splitter. ($T = 1/2$). **B**: measurement sensitivity for the same conditions in **A.**

$$\sigma^2(n_1)_{\text{on/off,SV}} = \frac{1}{\sqrt{1-\bar{n}_{\text{in}}(-2+T)T}}\left(1 - \frac{1}{\sqrt{1-\bar{n}_{\text{in}}(-2+T)T}}\right), \tag{S4}$$

$$\sigma^2(n_2)_{\text{on/off,SV}} = \frac{1}{\sqrt{1-\bar{n}_{\text{in}}(-2+R)R}}\left(1 - \frac{1}{\sqrt{1-\bar{n}_{\text{in}}(-2+R)R}}\right), \tag{S5}$$

$$\text{Cov}(n_1, n_2)_{\text{on/off,SV}} = \frac{1}{\sqrt{1+\bar{n}_{\text{in}}}} - \frac{1}{\sqrt{1+\bar{n}_{\text{in}}-\bar{n}_{\text{in}}\,R^2}\sqrt{1+\bar{n}_{\text{in}}-\bar{n}_{\text{in}}\,T^2}}. \tag{S6}$$

Here, T (R) is the transmittance (reflectance) of the beam splitter considering the input state port. The beam splitter is assumed to be lossless, $T + R = 1$. $\bar{n}_{\text{in}}$ is the average photon number of the input state. The variance of the differential measurement using a 50:50 beam splitter can be written as

$$\sigma^2(n^-)_{\text{on/off,SV}} = -\frac{2}{\sqrt{1+\bar{n}_{\text{in}}}} + \frac{4}{\sqrt{4+3\bar{n}_{\text{in}}}}. \tag{S7}$$

The standard deviation $\sigma(n^-) = \sqrt{\sigma^2(n^-)}$ of the differential measurement as a function of the input state average photon number $\bar{n}_{\text{in}}$, for different detection methods (PNR and on/off) and for coherent and squeezed vacuum input states is shown in Figure S2.

The on/off detection is characterized by a smaller standard deviation than the PNR detection. The squeezed vacuum state is overlapped with the coherent state in the case of PNR detectors. The squeezed vacuum state measured by on/off detectors shows the smallest standard deviation.

S.3. Detection sensitivity

The detected signal for each laser pulse is $\phi \simeq n^-/n^+$, where n^- and n^+ are the average number of photons per pulse in a differential measurement (Ch1-Ch2) and in the sum among the two channels (Ch1+Ch2), respectively. The sensitivity of the measurement can, therefore, be assessed by the signal-to-noise ratio:

$$SNR = \frac{\phi}{\sigma(\phi)} \simeq \frac{n^-}{\sigma(n^-)}. \tag{S8}$$

This ratio is shown in Fig. S2 B for the 4 cases considered in the previous section (PNR and on/off detection, squeezed vacuum and coherent input seed) as a function of $\bar{n}_{\text{in}}$. We have assumed to work in a condition where the detection is balanced, that is, for $T = R = 0.5$. It can be noted that the sensitivity is the same for the coherent and the squeezed vacuum input states in the case of PNR detection. The sensitivity for on-off detectors is, instead, lower for the squeezed vacuum case.

S.4. Statistical properties of the state realized in the experiments

As discussed in the main text, we have recorded the counts at the output of the beam splitter, Ch1 and Ch2, summed over periods of duration $T_s = 1/2f$, with $f = 10$ kHz. An example of the histogram for one of the two channels is shown in Fig. S3 A, obtained over a sampling time of 30 s (600,002 samples). The average number of counts in each integration time is $\bar{N}_i = 53.869$, and the average count rate on each of the two detectors is 1.077 MHz. We can, therefore, estimate the average number of counts per pulse, considering the 80 MHz laser repetition rate: $\bar{n}_i = 0.0135$. We employed the Strawberry Fields software [3,4] to simulate the measurement including $\eta = 0.65$ channel efficiency (losses due to detector efficiency and coupling).

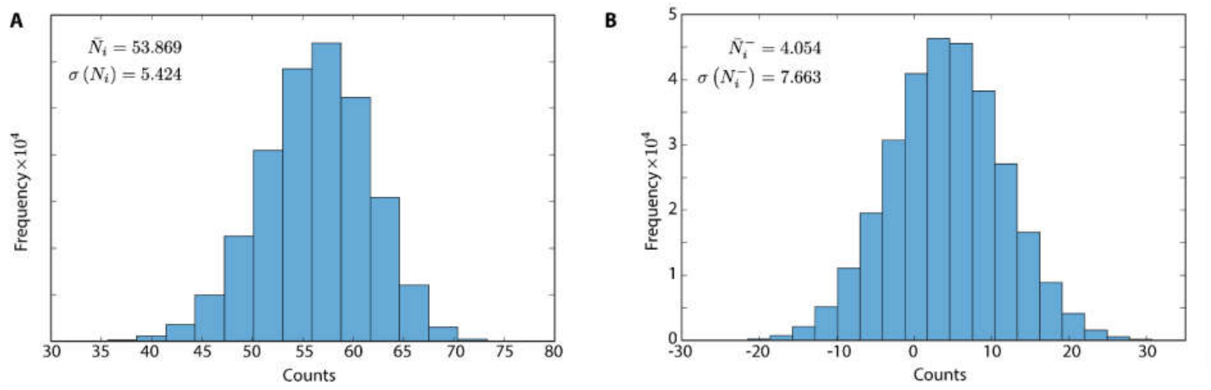

Figure S3: Experimental data statistical properties. **A**: Histogram of counts in Ch1 over a 30s sampling time. **B**: Histogram of the differential measurement Ch1-Ch2 over a 30s sampling time.

Considering a squeezed vacuum input state, an average number of photons per pulse of $\bar{n}_i = 0.0135$ is expected for an average photon number at the input of $\bar{n}_{\mathrm{SV}} = 0.0506$. The variance of the experimental data, $\sigma_{\mathrm{measured}}(n_i) = 0.0858$, is in good match with what is expected from the model, $\sigma_{\mathrm{model}}(n_i) = 0.0939$. Note that the model result differs from the prediction from Eq. S4 as the latter has been derived by neglecting channel losses.

In our measurement, we recorded the difference in counts between Ch1 and Ch2 for intervals where the THz field is present (or the electro-optical modulator is biased) and for intervals where the modulation is not present. An example of histograms for the same conditions mentioned before and for an active modulation is shown in Fig. S3 B. From these measurements we can compute the standard deviation of the distribution n^-, that is, the counts per pulse expected from the differential measurement Ch1-Ch2. We obtained $\sigma_{\mathrm{measured}}(n^-) = \sigma(N^-)/\sqrt{M} = 0.121$, where $M = 80\ \mathrm{MHz}/(2f) = 4000$ is the number of laser pulses in each acquisition interval. From the model, including losses, we predicted $\sigma_{\mathrm{model}}(n^-) = 0.126$. The good match between the model and the experimental data confirms that our measurements are limited in sensitivity by the statistical properties of the employed state. Also in this case, the results from the model differ from the prediction of Eq. S7 as the latter has been derived neglecting channel losses. According to the model, the average number of photon in the input squeezed vacuum state is $\bar{n}_{\mathrm{SV}} = 0.0506$.

We modeled the phase measurement in Strawberry Fields considering the effect of on/off detectors and losses. We first investigated the detection sensitivity according to Eq. S8 for lossy channels with $\eta = 0.65$. The results are shown in Fig. S4 A. The overall effect of losses is that of reducing the sensitivity with respect to the lossless case shown in Fig. S2 B for the same $\bar{n}_{\mathrm{in}}$. We have then assessed the value of $\alpha(\bar{n}_{\mathrm{SV}}, \eta)$ described in the main text, Eq. (4). Specifically, we computed:

$$\alpha = \frac{n^-_{\mathrm{SV,on/off}}}{n^+_{\mathrm{SV,on/off}}} \cdot \frac{n^+_{\mathrm{C,PNR}}}{n^-_{\mathrm{C,PNR}}} \qquad \text{(S9)}$$

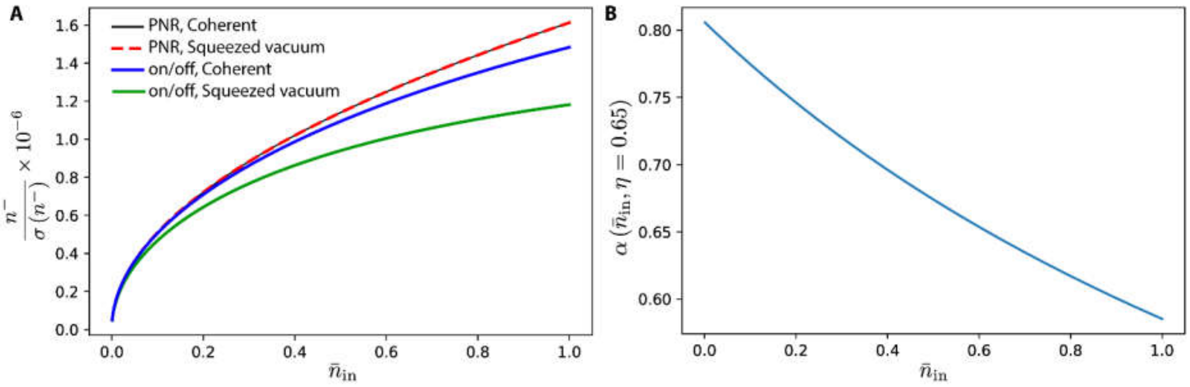

Figure S4: Theoretical analysis of the expected measurement sensitivity including losses. **A**: Measurement sensitivity as a function of average number of photons in the input state $\bar{n}_{\mathrm{in}}$ considering a channel with efficiency $\eta = 0.65$. **B**: Reduction parameter linking the measured phase to the effective one for a squeezed vacuum input state, on/off detectors and $\eta = 0.65$ channel efficiency.

for $\eta = 0.65$ and as a function of $\bar{n}_{\mathrm{in}}$. The result is shown in Fig. S4 B. From this analysis, in our experimental condition ($\bar{n}_{\mathrm{in}} = \bar{n}_{\mathrm{SV}} = 0.0506$) we obtained $\alpha = 0.790$.

Supplementary references

1. de Araújo, M.A.; Silva, S.; de Lima, W.; Pereira, D. P.; de Oliveira, P. C. Measurement of Gaussian laser beam radius using the knife-edge technique: improvement on data analysis. *Appl. Opt.* **2009**, 48, 393-396. https://doi.org/10.1364/AO.48.000393.
2. Alléaume, R.; Treussart, F.; Courty, J.M.; Roch, J.F. Photon statistics characterization of a single-photon source. *New J. Phys.* **2004**, *6*, 85. https://doi.org/10.1088/1367-2630/6/1/085.
3. Killoran, N.; Izaac, J.; Quesada, N.; Bergholm,V.; Amy, M.; Weedbrook, C. Strawberry Fields: A Software Platform for Photonic Quantum Computing. *Quantum*, **2019**, 3, 129. https://doi.org/10.22331/q-2019-03-11-129.
4. Bromley, T. R.; Arrazola, J. M.; Jahangiri, S.; Izaac, J.; Quesada, N.; Gran, A. D.; Schuld, M.; Swinarton, J.; Zabaneh, Z.; Killoran, N. Applications of Near-Term Photonic Quantum Computers: Software and Algorithms. *Quantum Sci. Technol.* **2020**, 5, 034010, https://doi.org/10.1088/2058-9565/ab8504
